# Analysis of the anticancer activity of curcuminoids, thiotryptophan and 4-phenoxyphenol derivatives

**DOI:** 10.3892/ol.2013.1679

**Published:** 2013-11-12

**Authors:** SHIREEN PARSAI, RICK KECK, EWA SKRZYPCZAK-JANKUN, JERZY JANKUN

**Affiliations:** 1 Department of Urology, Urology Research Center, College of Medicine, University of Toledo, Toledo, OH 43614, USA; 2 Protein Research Chair, Department of Biochemistry, College of Sciences, King Saud University, Riyadh 11451, Kingdom of Saudi Arabia; 3 Department of Clinical Nutrition, Medical University of Gdańsk, Gdańsk 80-211, Poland

**Keywords:** curcumin, thiotryptophan, 4-phenoxyphenol, derivatives, anticancer

## Abstract

Curcumin, a non-nutritive yellow pigment derived from the rhizome of *Curcuma longa* (turmeric), is considered to be an established nutraceutical with anticancer activity. Turmeric contains three principal components, curcumin, demethoxycurcumin and bisdemethoxycurcumin, of which curcumin is most abundant and potent. The concurrence of a high consumption of turmeric and a low incidence of prostate cancer in Asian countries may suggest a role for curcumin in chemoprevention. Curcumin has been identified to exhibit anti-inflammatory, anti-oxidative and anticarcinogenic properties. Since the compound does not exhibit side effects, curcumin has been designated for several clinical trials as a treatment for human cancers. The pro-apototic, antioxidant and anti-inflammatory characteristics of curcumin are implicated in its anticancer activity, yet the mechanism of action of curcumin remains unknown. To achieve an effective pharmacological outcome, curcumin must reach and sustain appropriate levels at the site of action. However, the main disadvantage of curcumin is its high metabolic instability and poor aqueous solubility that limits its systemic bioavailability. To overcome this difficulty, the present study tested the anticancer activity of new curcumin-like compounds (E21cH and Q012095H). Also, the use of new medicaments requires an understanding of their pharmacokinetic profiles and targets. Thus, molecular modeling methods were used to identify the targets of curcumin and curcumin-like compounds compared with other anticancer drugs (Q012138 and Q012169AT), which were used as the controls. The present study identified several enzymes that are targeted by curcumin, aldo-keto reductase family 1 member B10 (AKR1B10), serine/threonine-protein kinase, protein kinase C, matrix metalloproteinase (MMP), cyclooxygenase and epidermal growth factor receptor, which were tested as targets for these anticancer chemicals. All the examined small compounds demonstrated anticancer activity in the *in vitro* experiments and may impact cancer cells by acting on AKR1B10, MMP-9 and their targets.

## Introduction

According to the American Cancer Society, prostate cancer is currently the second most common cause of cancer-related mortality among males. An estimated >235,000 new cases of prostate cancer are expected in the US during 2013 (1). Furthermore, in a recent study by Arcangeli *et al*, it is predicted that the increase in birth rate may correlate with an increased prevalence of prostate cancer in the United States by 2020 (2). However, with the establishment of diagnostic markers, including prostate-specific antigen screening, and recent advances in molecular imaging, clinicians are able to detect early cancer proliferation prior to the development of apparent clinical manifestations and, more significantly, prior to the occurrence of metastasis. This affords clinicians more time to design the appropriate and effective treatment procedures. The current treatment methods for prostate cancer include the administration of steroidal and non-steroidal anti-androgens, radiation therapy, chemotherapy, surgery or a combination of these modalities. Although these options may be successful in controlling the progression of prostate cancer, they are often associated with comorbidities that affect urinary and sexual function. Therefore the aim of prostate cancer research is to develop innovative treatment options to avoid such complications. Several characteristics of prostate cancer make it useful to serve as a model for developing new chemopreventive techniques, including its high prevalence, heterogeneous presentation, long latency, slow progression, preneoplastic lesions and tumor marker availability (2,3).

Males have an equal rate of histological prostate cancer worldwide, as assessed by volume, grade and number of malignant foci (4). However, disease incidence varies widely according to the geographic location. Western nations have higher rates of mortality compared with Asian countries, including India, China and Japan. More notably, migrating populations from low-risk areas (Asian countries) to high-risk areas (Western countries) also have an increased risk of developing prostate cancer. Since genetic predisposition accounts for only 5–10% of cases, as cited by the American Cancer Society (1), the uniting theme in the literature has become identifying the environmental factors that promote or inhibit the development of prostate cancer.

Foods or part of foods with medicinal value, termed nutraceuticals, which are prepared and consumed variably across cultures, may be active in the prevention and treatment of diseases, including prostate cancer. Curcumin, a non-nutritive yellow pigment derived from the rhizome of *Curcumin longa* (turmeric), has received attention as an established nutraceutical that is capable of anticancer activity (5). Turmeric contains three principal components, curcumin, demethoxycurcumin and bisdemethoxycurcumin, of which curcumin is the most abundant and potent (6–9). The concurrence of a high consumption of turmeric in Asian countries and a low incidence of prostate cancer suggest its role in chemoprevention (10). Curcumin and a number of its derivatives have been identified to exhibit anti-inflammatory, antioxidative and anticarcinogenic properties (11). As the compound does not exhibit toxic, genotoxic or teratogenic properties, curcumin has been selected for several clinical trials to be used as a possible treatment for human cancers (3,5,11). Curcumin has been shown to diminish the proliferation of androgen-dependent and androgen-independent prostate cancer cell lines (12). Furthermore, studies have revealed a wide array of therapeutic activities against multiple myeloma, pancreatic cancer, myelodysplastic syndromes, colon cancer, psoriasis, Alzheimer’s disease and others (13). The pro-apototic, antioxidant and anti-inflammatory properties of curcumin are implicated in its anticancer activity, yet the mechanism of action of curcumin remains unknown (8). Curcumin is a highly pleiotropic molecule with multiple mechanisms by which it may mediate chemotherapy and chemopreventive effects on cancer, while remaining safe with little or no side effects. This dietary compound has been shown to inhibit several cell signaling pathways, including nuclear factor (NF)-κB, activating protein-1, tumor necrosis factor and metastatic and angiogenic pathways. The compound also inhibits certain enzymes, including cyclooxygenase (COX)-2 and matrix metalloproteinases (MMPs) (9,13,14). The present study randomly identified several enzymes that are essential in carcinogenesis and are also targeted by curcumin, aldo-keto reductase family 1 member B10 (AKR1B10), serine/threonine-protein kinase (mTOR), protein kinase C (PKC), MMP-9, COX-1 and epidermal growth factor receptor (EGFR), to gain further insight into the mechanism of action (5,7,13,15–17).

Curcumin has a poor systemic bioavailability as it is not able to reach and sustain the appropriate levels at the site of action due to its high metabolic instability and poor aqueous solubility (18,19). The present study aimed to identify the anticancer activity of curcumin-like compounds with potentially greater bioavailability, and speculate the protein targets of these compounds that are implicated in the mechanism of action. Novel curcumin-like compounds, E21cH and Q012095H, with greater water solubility were tested. Molecular modeling methods were used to identify the targets of curcumin and curcumin-like compounds by comparing them with other anticancer drugs (Q012138 and Q012169AT), which were used as a controls.

## Materials and methods

### Compounds

**Figure 1:**
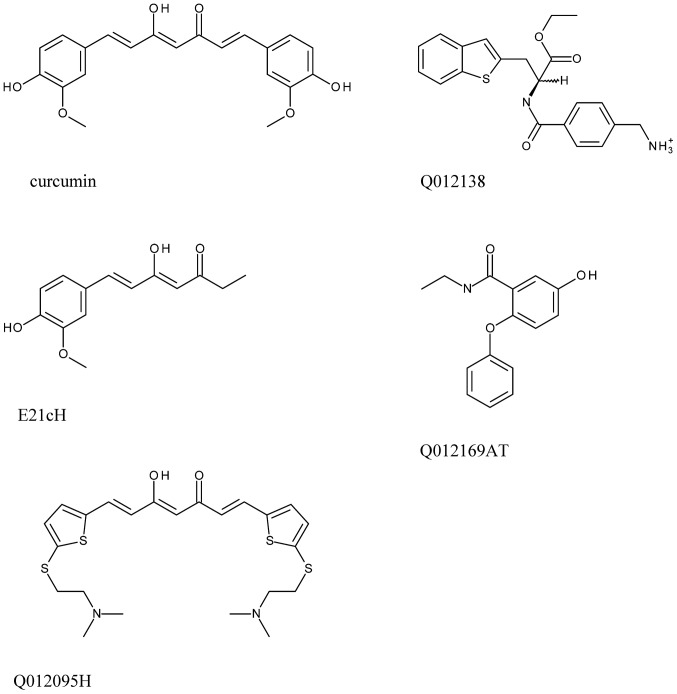
Structure of compounds with anticancer activities. Curcumin and curcumin-like compounds are presented in the enol form, which is more thermodynamically stable (60).

The small molecular chemicals with anticancer activities were obtained from PharmaIP, LLC (Greenwich CT, USA). Curcumin [(1E,4Z,6E)-5-hydroxy-1,7-bis(4-hydroxy-3-methoxy-phenyl)hepta-1,4,6-trien-3-one]; Q0121138 [4-[[(1S)-1-(benzothiophen-2-ylmethyl)-2-ethoxy-2-oxo-ethyl]carbamoyl]phenyl] methylammonium; Q012095H (1E,4Z,6E)-1,7-bis[5-(2-dimethylaminoethyl sulfanyl)-2-thienyl]-5-hydroxy-hepta-1,4,6-trien-3-one; Q012138 [4-[[(1S)-1-(benzothiophen-2-ylmethyl)-2-ethoxy-2-oxo-ethyl]carbamoyl] phenyl] methylammonium; and Q012169AT (N-ethyl-5-hydroxy-2-phenoxy-benzamide; Fig. 1). All the compounds were dissolved in dimethyl sulfoxide (DMSO) 2.5 mg/ml and stored at −20°C until they were used.

### Cell culture and clonal assay

The DU-145 human prostate cancer cell line was grown in RPMI-1640 medium supplemented with 10% fetal bovine serum (Atlanta Biologicals, Lawrenceville, GA, USA) and 100 U/ml penicillin, 100 μg/ml streptomycin (Sigma-Aldrich, St. Louis, MO, USA). A total of ~50 or ~100 viable DU-145 cells (Trypan blue viability assay, two separate trials) were plated in 0.5 or 1 ml of complete medium onto 12 or 24-well tissue culture dishes. The cells were allowed to attach for 48 h. The cells were then treated for 4 h with 1.2-, 2.5-, 5.0- or 10-μl allotments of DMSO, curcumin, E21cH, Q0121138, Q012095H or Q012169AT dissolved in 1 mg/ml DMSO. The surviving cells were incubated for nine days to allow colony formation and then rinsed with 10% saline, fixed with 100% methanol and stained using Giemsa stain. The colony counts were performed under ×10 magnification (Stereomaster, Thermo Fisher Scientific Inc., Waltham, MA, USA). The experiments were repeated in triplicate to determine the anticancer activity.

### Molecular modeling

Two-dimensional structures (2D) of small molecular chemicals were created by AccelrysDraw v. 4.0 (Accelrys, Inc., San Diego, CA, USA) in an SKC format. The 2D structures were converted into three dimensional and PDB format files using a web-based program (http://www.molecular-networks.com/products). Docking of the potential inhibitors to the proteins was performed using VINA Autodock (Molecular Graphics Lab, The Scripps Research Institute, La Jolla, CA, USA) (20). The protein structures were downloaded from http://www.rcsb.org/pdb/home/home.do as: 1zua, AKR1B10 (21); 3oaw, mTOR (22); 1yrk, PKC (23); 2ovx, MMP-9 (24); 3ln1, COX-2 (25) and 2itx, EGFR (26). A search box was set up with following parameters: AKR1B10 human NADPH-dependent aldo-keto reductase (center: x, −29; y, 22; z, 0.1; size: x, 50; y, 50; z, 50), mTOR (center: x, −17.5; y, −11; z, −12; size: x, 50; y, 40; z, 46). PKC (center: x, 25; y, 40; z, 31; size: x,40; y, 44; z, 74), MMP-9 (center: x, 27; y, 6; z, 51; size: x, 40; y, 56; z, 40), COX-2 (center: x, 32; y, −22; z, −16; size: x, 40; y, 40; z, 40) and EGFR (center: x, −47; y, −2; z, −22; size: x, 50; y, 40; z, 50). The inhibitors that were present in the PDB structures were used to determine the center of the search and later removed from structure. The small molecules were kept flexible by allowing rotation around the single bonds. By default, VINA Autodock analyzes eight various protein/inhibitor complexes (conformers) and the one with the lowest free energy is considered the most probable. Free energy is converted to K_i_ using the following formula (20,27–30): K_i_ = exp [ΔG / (R × T)], where ΔG is Gibbs free energy change, R is the gas constant and T is the absolute temperature. The final analyses of structures that were generated by Autodock and the generation of the figures was performed using PyMOL v. 1.4 (Schrödinger, München, Germany) (31,32).

## Results

### In vitro anticancer activity

**Figure 2:**
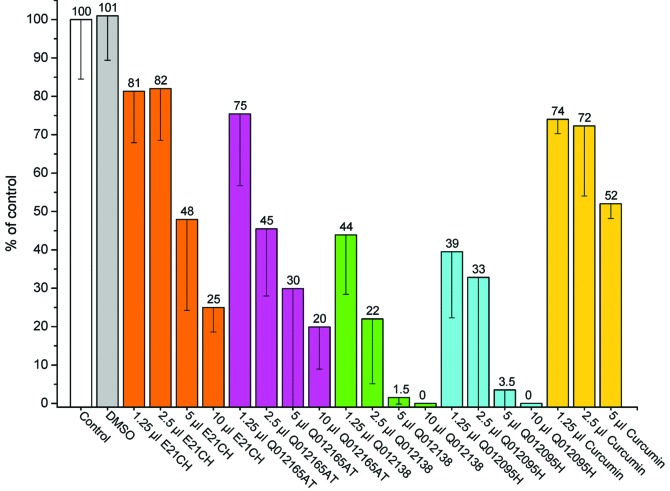
Survival of cancer cells treated with the chemicals that were tested depends on the concentration of the delivered compound in the cell media. DMSO, dimethyl sulfoxide.

The present study tested the anticancer activities of >30 curcuminoids, thiotryptophanes and 4-phenoxyphenol derivatives. In the clonal assay, Q012095H demonstrated the strongest anticancer activity, followed by Q012138 and Q012165H. E21cH and curcumin activity were comparable with each other but lower than the three others (Fig. 2). The highest concentrations of Q012095H and Q012138 showed a complete inhibition of cancer cell growth.

### Molecular modeling

**Figure 3:**
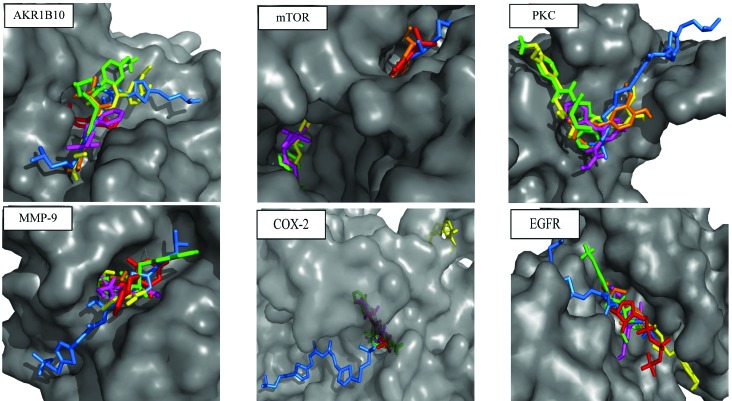
Surface model of the active sites of the enzymes that were tested. All the potential inhibitors are shown as stick models. Curcumin (yellow), E21cH (orange), Q012095H (blue), Q012138 (green) and Q012169AT (magenta). The inhibitor from the PDB structure is shown in red. AKR1B10, aldo-keto reductase family-1 member B10; mTOR, serine/threonine kinase; PKC, protein kinase C; MMP, matrix metalloproteinase; COX-2, cyclooxygenase-2; EGFR, epidermal growth factor receptor.

The results of the docking are illustrated in Fig. 3 and the calculated K_i_ values are provided in Table I. All the compounds that were tested contained an aldo-keto moiety. One of the human enzymes that was tested in the *in silico* experiment was AKR1B10, an NADPH-dependent aldo-keto reductase that reduces a variety of aldehydes and ketones. AKR1B10 has been reported to be upregulated in number of cancers. Additionally, AKR1B10-gene silencing results in the inhibition of colorectal cancer cell growth, suggesting that AKR1B10 regulates cell proliferation (33). It has been proposed that AKR1B10 controls retinoic acid signaling and impacts the carcinogenesis process. Also, tolrestat, which efficiently inhibits AKR1B10, is suggested to have a potential application in cancer control (34,35). Thus, the investigated chemicals were tested for the capacity to bind to the active site of this enzyme. All the investigated chemicals were found to bind near the active site and functionally block its access. The calculated K_i_ was in μM levels for all the tested chemicals, indicating their relative strength of affinity (Table I).

## Discussion

mTOR, a serine/threonine protein kinase, regulates cell growth, cell proliferation, cell motility, cell survival, protein synthesis and transcription (36). The inhibition of mTOR mediates the antiproliferative effects of curcumin in numerous human and non-human cell lines (15,37,38). In addition, curcumin has been reported to be able to dissociate the raptor subunit from mTOR as well as inhibit mTORC1 activity (15). Liu *et al* designed several idopyrimidinone (1) 4-methylpteridinones that bind to a small pocket within the mTOR binding site. This inhibitor in the protein structure was used as a center of search (22). In the present study, the molecular modeling revealed that all the compounds that were tested had a relatively low affinity and bound in various locations outside the active site of mTOR. Only E21cH and Q012095H were able to bind in proximity to where the inhibitor was localized. mTOR may be an unlikely target of the chemicals that were tested. By contrast, Lin *et al* stated that PKC and mTOR were the major upstream molecular targets for curcumin (39). A possible explanation is that the products of curcumin degradation act on mTOR instead of curcumin itself (7,8,13,14,18,19,40).

Curcumin is an inhibitor of PKC. Consequently, curcumin inhibits the activation of NF-κB and the expression of oncogenes, including c-jun, c-fos, c-myc, NF-κB-inducing kinase (NIK), mitogen-activated protein kinases, ERK, ELK, phosphoinositide 3-kinase, Akt, cyclin-dependent kinases and inducible nitric oxide synthase (39). Conboy *et al* performed molecular modeling and identified that curcumin was able to dock effectively on PKC. However, curcumin did not directly inhibit PKC activity, but rather increased its degradation (41). The calculations in the present study revealed all tested compounds bind to PKC in essentially the same place, but with low affinity.

Traditionally, MMP-9 was associated with tumor angiogenesis and metastasis by lysing proteins of connective tissue (42,43). However, curcumin has been reported to protect MMP-9 from proteolytic degradation (44). MMP-9 plays a critical function in normal and pathological angiogenesis and/or controlling the biological activity of growth factors, cytokines and chemokines (45). Proteolytic enzymes that stimulate angiogenesis and metastasis frequently show other functions in carcinogenesis in addition to their traditional roles (46–48). Ravindranath *et al* reported that blocking the activity of MMP-9 may arrest cell growth and proliferation in addition to the inhibition of invasion and angiogenesis (49). All the chemicals that were tested were observed to bind to the active site of MMP-9 in the proximity of the MMP-9 inhibitor (5-(4-phenoxy phenyl)-5-(4-pyrimidin-2-ylpiperazin-1-yl)pyrimidine-2,4,6(2H,3H)-trione) with high affinity.

Curcumin possesses anti-inflammatory activity and is a potent inhibitor of reactive oxygen generating enzymes, including COX (15,37). Curcumin itself is a potent scavenger of free radicals and the inhibition of COX potentiates its anticancer activity (50–52). Although the specific regulation of COX-2 by curcumin is not fully understood, the evidence suggests that curcumin regulates COX-2 at the transcriptional and the post-translational levels (17,53). In the present study, molecular modeling revealed that E21cH, Q012138 and Q012169AT bind to COX-2 with a high affinity, deep in the tunnel of the active site where celecoxib (4-[5-(4-methylphenyl)-3-(trifluoromethyl)-1h-pyrazol-1-yl]benzenesulfonamide) is bound (25). However, Q012095H and curcumin were observed to bind outside of this site. This is in contrast with studies that state that curcumin inhibits COX (16,54–56). A possible explanation is that the non-enzymatic degradation of curcumin occurs, resulting in degradation products that are formed through cleavage of the heptadienone chain that connects the phenolic rings (57). Dong *et al* have shown that COX acts as a dimer, where one monomer with a heme moiety is active and the other is apo, which acts as the allosteric site, controlling activity of the active monomer (58). Thus, the docking scenario in its simplification *in silico* may not reflect the true situation *in vivo*, which may be more complex.

Xu *et al* reported that cyclohexanone analogs that are designed based on the curcumin structure are potential EGFR inhibitors and exhibit antiproliferative activity in human tumor cell lines. Cyclohexanone analogs fit in the active site of EGFR, as shown by molecular docking (59). This was confirmed by the experimental modeling in the present study. All the investigated compounds also bound the EGFR active site, but with low affinities.

Based on the results of the present study, AKR1B10 and MMP-9 have been shown to be the most likely targets of curcumin and curcumin-like derivatives. Curcumin and the investigated curcumin-like compounds bound the other proteins that were tested outside of the active site or with low affinities.

## Figures and Tables

**Table I:** Calculated K_i_ for the various protein and inhibitor complexes.

Compound	AKR1B10 (kcal/M)/K_i_(M)	mTOR (kcal/M)/K_i_(M)	PKC (kcal/M)/K_i_(M)	MMP-9 (kcal/M)/K_i_(M)	COX-2 (kcal/M)/K_i_(M)	EGFR (kcal/M)/K_i_(M)
Curcumin	−7.6/2.8×10^−6^	−6.9/9.0×10^−6^ NA	−6.2/2.9×10^−5^	−9.1/2.2×10^−7^	−7.3/4.6×10^−6^ NA	−6.7/1.3×10^−5^
E21cH	−7.4/3.9×10^−6^	−5.8/5.7×10^−5^	−4.9/2.6×10^−4^	−7.9/1.7×10^−6^	−7.6/2.8×10^−6^	−6.3/2.5×10^−5^
Q012095H	−5.8/5.7×10^−5^	−4.6/4.3×10^−4^	−4.5/5.1×10^−4^	−6.2/2.9×10^−5^	−6.1/3.5×10^−5^ NA	−5.2/1.6×10^−4^
Q012138	−7.1/6.5×10^−6^	−6.2/2.9×10^−5^ NA	−5.8/5.7×10^−5^	−8.9/3.1×10^−7^	−9.2/1.9×10^−7^	−6.2/2.9×10^−5^
Q012169AT	−6.2/2.9×10^−5^	−6.9/9.0×10^−6^ NA	−4.9/2.6×10^−4^	−7.8/2.0×10^−6^	−8.8/3.7×10^−7^	−6.6/1.5×10^−5^

AKR1B10, aldo-keto reductase family-1 member B10; mTOR, serine/threonine kinase; PKC, protein kinase C; MMP, matrix metalloproteinase; COX-2, cyclooxygenase-2; EGFR, epidermal growth factor receptor; NA, not applicable.
